# Deep Learning-Based Imbalanced Classification With Fuzzy Support Vector Machine

**DOI:** 10.3389/fbioe.2021.802712

**Published:** 2022-01-21

**Authors:** Ke-Fan Wang, Jing An, Zhen Wei, Can Cui, Xiang-Hua Ma, Chao Ma, Han-Qiu Bao

**Affiliations:** ^1^ School of Electrical and Electronic Engineering, Shanghai Institute of Technology, Shanghai, China; ^2^ School of Design, East China Normal University, Shanghai, China; ^3^ College of Electronic and Information Engineering, Tongji University, Shanghai, China

**Keywords:** imbalance classification, deep neural network, fuzzy support vector machine, machine learning, oversampling

## Abstract

Imbalanced classification is widespread in the fields of medical diagnosis, biomedicine, smart city and Internet of Things. The imbalance of data distribution makes traditional classification methods more biased towards majority classes and ignores the importance of minority class. It makes the traditional classification methods ineffective in imbalanced classification. In this paper, a novel imbalance classification method based on deep learning and fuzzy support vector machine is proposed and named as DFSVM. DFSVM first uses a deep neural network to obtain an embedding representation of the data. This deep neural network is trained by using triplet loss to enhance similarities within classes and differences between classes. To alleviate the effects of imbalanced data distribution, oversampling is performed in the embedding space of the data. In this paper, we use an oversampling method based on feature and center distance, which can obtain more diverse new samples and prevent overfitting. To enhance the impact of minority class, we use a fuzzy support vector machine (FSVM) based on cost-sensitive learning as the final classifier. FSVM assigns a higher misclassification cost to minority class samples to improve the classification quality. Experiments were performed on multiple biological datasets and real-world datasets. The experimental results show that DFSVM has achieved promising classification performance.

## Introduction

In many fields, the distribution of data is imbalanced and the problem of imbalanced datasets occurs when one class is much larger than the other. For example, in disease diagnosis (Bhattacharya et al., 2017; Loey et al., 2020), most of the data are healthy, and it is difficult to obtain data on diseases. Moreover, with the deployment of various monitoring systems, more and more data are collected in smart cities and the Internet of Things, but there are a lot of data on the normal operation and abnormal data is rare (Du et al., 2019; Fathy et al., 2020). More specifically, this problem occurs when one class outnumbers the other class, which are usually referred to as majority and minority class, respectively (Tao et al., 2020). The majority class samples are more easily available, while the minority class samples are more difficult to obtain data due to natural frequency of occurrence or data collection. The imbalanced data distribution also exists in the fields of fraud detection (Li and Wong, 2015; Jiang et al., 2018), computer security (Wang and Yao, 2013), intrusion detection (Yao et al., 2018), drift detection (Wang et al., 2021), image recognition (Romani et al., 2018) and defect detection (Li et al., 2020). In machine learning, there are many well-established classification methods, such as decision tree, logistic regression, support vector machine and extreme learning machine (Shi et al., 2022), but they are based on the assumption of uniform data distribution and have over-all accuracy as the optimization goal. When traditional classification methods are used to deal with imbalanced classification, the result are more in favor of the majority class and ignore the importance of the minority class. Although the overall accuracy is relatively high, the minority class data with important information cannot be accurately identified.

Many imbalance classification algorithms have been proposed in recent decades. These algorithms can be generally divided into two main types: data-level and algorithm-level (Tao et al., 2020). The data-level approaches first bring the original imbalanced dataset to balanced distribution by some sampling processing, and then classify it by using a traditional classifier. The algorithm-level approaches attempt to improve existing classification algorithms by reducing their bias for the majority class data, and thus adapt to imbalanced data distribution.

In this paper, a novel imbalance classification method based on deep feature representation is proposed, named DFSVM. First, from the perspective of data features, a deep neural network is used to obtain the embedding space features. Appropriate feature representation can improve the classification performance of models, and it also enhances the differentiation of features of different classes and the similarity of feature areas of the same class. In addition, it will provide a basis for the effective recognition of samples. The deep neural network has a complex nonlinear network structure, which can effectively extract the deep features of samples. When training the network, a triplet loss function (Schroff et al., 2015) is used to enable the network to separate the features of minority class and majority class. Additionally, Gumbel distribution function (Cooray, 2010) is applied as an activation function in the activation layer. This function is continuously differentiable, and it can be easily used as an activation function in stochastic gradient descent optimization neural networks. The original input samples are mapped to the same embedding space after feature extraction. In the embedding space, a new minority class sample is randomly generated based on the distance between the sample and the center of the class, which makes the data distribution balanced. After obtaining the embedding features of samples, a fuzzy support vector machine (FSVM) (Lin and Wang, 2002) is used to classify. FSVM introduces membership values (MVs) in the objective function of traditional support vector machine, and it sets different misclassification costs for different classes samples. The misclassification cost of the minority class is higher than that of the majority class. FSVM is a cost-sensitive learning strategy that can effectively improve the recognition rate of the minority class samples. In addition, traditional classification methods use accuracy as classifier evaluation metrics, but classifiers with accuracy as evaluation metrics tend to reduce the classification effectiveness of the minority class. Moreover, accuracy limits the effect of minority class samples on classification performance. Therefore, this paper uses G-mean, F-measure and AUC values to evaluate the classification results more comprehensively.

The rest of this paper is organized as follows. In *Related Work* Section, the related work on imbalance classification is presented. *Proposed Method* Section describes DFSVM. In *Experiments and Results and Conclusion* Sections, the experimental results and conclusions are introduced.

## Related Work

The imbalance of data distribution and the limitation of traditional classification algorithms are the main problems that imbalanced classification faces, therefore, researches on imbalanced classification can be divided into two levels: data-level and algorithm-level.

### Data-Level

Data resampling is the most representative method of data-level, which reduces the imbalanced ratio (IR) by changing the data distribution. The undersampling algorithm reduces the bias of model to the majority class by reducing the number of samples in the majority class. Random undersampling is the simplest approach, it randomly selects and removes part of the majority class samples. However, random undersampling easily leads to the deletion of potentially useful information, so some heuristic methods are proposed.

Neighborhood cleanup rule (NCL) (Laurikkala, 2001) uses an instance-based approach to reduce larger classes and considers carefully the quality of the data to be removed. To reduce the impact of some noisy minority examples on the performance of classifiers, Kang et al. (2016) proposed a new undersampling algorithm by introducing a noise filter. The weighted under-sampling of SVM (WU-SVM) groups majority samples into some subregions and assigns different weights based on their Euclidean distance to the hyper plane to retain the data distribution information of original dataset (Kang et al., 2017). The other popular sampling method is oversampling, which is used to balance the data distribution by increasing the number of minority class samples. Random oversampling can cause overfitting, so heuristic methods are also mostly used. The most representative one is the synthetic minority oversampling technique (SMOTE, Chawla et al., 2002). SMOTE generates a new minority sample by interpolating between k nearest minority neighbors. However, due to the irregular data distribution, new samples generated by SMOTE may become noise, which may increase the overlap between classes and lead to misclassification. In order to generate more reasonable samples, some variants of SMOTE have been proposed, such as Bordeline-SMOTE (B-SMOTE) (Han et al., 2005) and adaptive synthetic sampling approach (ADASYN) (He et al., 2008). The kernel-based SMOTE (KSMOTE) algorithm synthesizes minority data points directly in the feature space of SVM classifier and adds new data points by augmenting the original Gram matrix based on neighborhood information in the feature space (Mathew et al., 2015). Weighted kernel-based SMOTE (WK-SMOTE) overcomes the limitations of SMOTE for nonlinear problems by oversampling in the feature space and cost-sensitive support vector machine (Mathew et al., 2018).

### Algorithm-Level

Traditional classification methods tend to favor majority class and ignore minority class samples when dealing with imbalanced data. To overcome the shortcomings of traditional classification, researchers have made improvements to the algorithms themselves. Typical improvements are cost-sensitive and ensemble learning methods. Fuzzy support vector machine (FSVM) (Lin and Wang, 2002) is a cost-sensitive algorithm. It introduces the fuzzy membership values (MVs) of each sample into the objective function of the support vector machine (SVM) to distinguish the importance of different samples. FSVM-CIL is an improved FSVMs for class imbalance learning that can be used to deal with class imbalances in the presence of outliers and noise, and its membership calculation is based on the distance in the original data space (Batuwita and Palade, 2010). Yu et al. (2019) proposed two relative density-based FSVM, namely, FSVM-WD based on within-class relative density and FSVM-BD based on between-class relative density, which use a similar strategy to calculate the relative density of each training sample based on K-nearest neighbor probability density estimation (KNN-PDE). ACFSVM is a FSVM method based on affinity and class probability, which calculates the affinity of majority class samples based on the support vector description domain (SVDD) model, and then identifies possible outliers and some border samples existing in the majority class (Tao et al., 2020). The basic idea of ensemble learning is to combine standard ensemble learning algorithms with existing imbalanced data classification methods, such as SMOTEBagging (Wang and Yao, 2009) and SMOTEBoost (Chawla et al., 2003). However, the training process of ensemble learning for base classifiers is more complicated and has limitations in handling high-dimensional data, and there are difficulties in choosing the type and number of base classifiers.

## Proposed Method

The DFSVM method proposed in this paper uses a fuzzy support vector machine as the base classifier and uses data sampling method to obtain balanced data distribution. The new samples generated after oversampling still belong to the minority class, and the use of FSVM can further improve the model’s focus on the minority class. In addition, deep neural networks are used to obtain more discriminative feature information, which make subsequent classification convenient.

### Feature Extraction With Deep Learning

With the significant increase in computer computing power and the explosive growth of data amount, deep learning has attracted a lot of attention in academia and industry in recent years. Deep neural networks (DNNs) have succeeded in significantly improving the best recognition rate of each previous problem by increasing the network depth or changing the structure of the model (Krizhevsky et al., 2012; He et al., 2016). Deep learning implementations rely on deep neural networks, which involves a cascade of multiple layers of nonlinear processing units for feature extraction and transformation. For example, convolutional neural networks (CNNs) are one such deep learning architecture that has achieved breakthrough performance gains in image classification (Kang et al., 2021). Feature representation is critical to the classification performance, so this paper applies the classification method to the embedding space after feature extraction.

In this paper, a deep neural network (DNN) is used as feature extractors because it can learn advanced feature representations from samples (Ng et al., 2016). Once training is complete, the hidden feature representations can be used as embedding features to reveal interesting structures in the data. To enhance the differentiation of features from different classes and reduce the differentiation of features from samples in the same class, a triplet loss (Schroff et al., 2015) is used to train the network model, bring samples in the same class closer, and further separate samples in different classes. Each sample can be converted into a differentiated feature space based on the trained model. The triplet loss uses anchor points, which allows the embedding space feature to be arbitrarily distorted. It is defined as:

$$\begin{array}{c} L_{\mathrm{triplet}}={\left(D_{a,\min}-D_{a,\mathrm{maj}}+m\right)}_{+} \end{array}$$ (1)

where *m* is the margin and set to 0.2 in experiments, *D* is the distance function, a is the anchor point belonging to the minority class, min is the minority class samples, and *maj* is the majority class samples. ${\left(\cdot \right)}_{+}$ indicates that the value is taken as loss if it is greater than 0. If it is less than 0, the loss is 0. The smaller the margin, the easier the triplet loss converges to 0. Thus, the anchor point and minority class samples do not need to be pulled too close together, and the anchor point and majority class samples do not need to be pulled too far apart to make the loss converge quickly to 0. However, the smaller margin cannot distinguish the similar samples well. When the margin is too large, the distance between the anchor point and the minority class samples needs to be desperately close, and the distance between the anchor point and the majority class samples needs to be faraway. If the margin is set too large, it is likely that the final loss will remain at a large value, which is difficult to converge to 0, but can distinguish more similar samples with more certainty.

**FIGURE 1:**
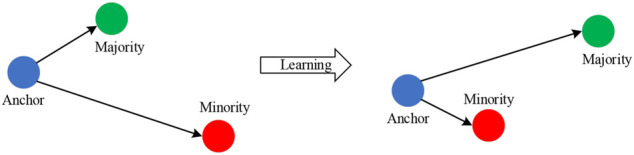
Optimization result using triple loss function.

Figure 1 shows the results and geometric significance of optimization using triple loss. Triplet loss tries to learn an embedding space in which anchor is closer to the minority class samples, and the anchor is further away from the majority class samples. The deep neural network model with the triplet loss as the training criterion not only takes the simplicity of metric learning into account, but also has excellent nonlinear modeling capabilities of neural networks, which can greatly simplify and control the training process. When the two inputs are similar, the triplet loss can learn a better representation for the two input vectors with smaller differences, and thus perform well in the classification task.

Gumbel distribution (Cooray, 2010) is used as the activation function in DNN. The Gumbel distribution, also known as Generalized Extreme Value (GEV) distribution type I, is widely used to design the distribution of extreme value samples of various distributions. The cumulative distribution function (CDF) is defined as:

$$\begin{array}{c} \sigma \left(x\right)=e^{-e^{-x}} \end{array}$$ (2)

**FIGURE 2:**
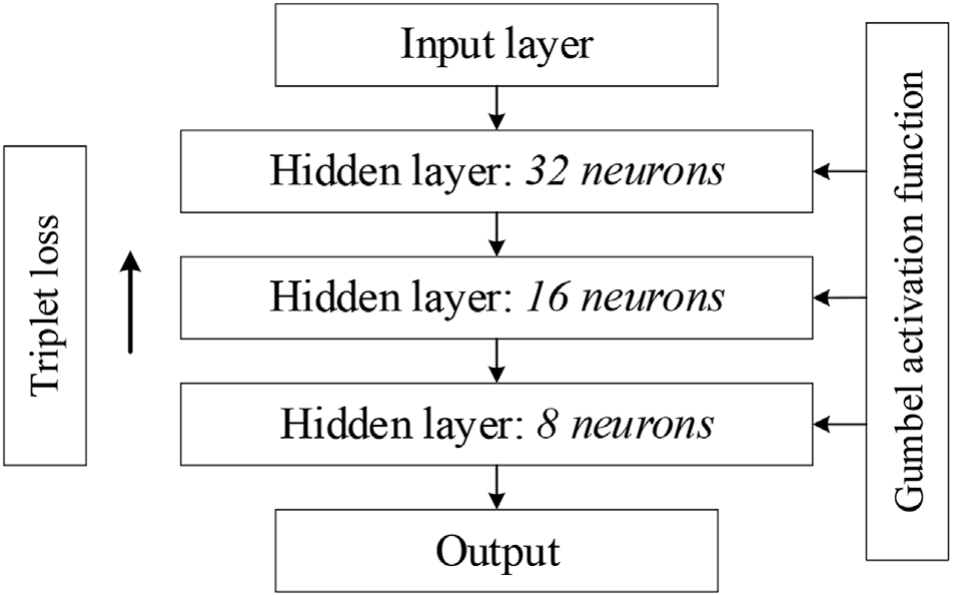
Deep neural network framework for feature extraction.

**FIGURE 3:**
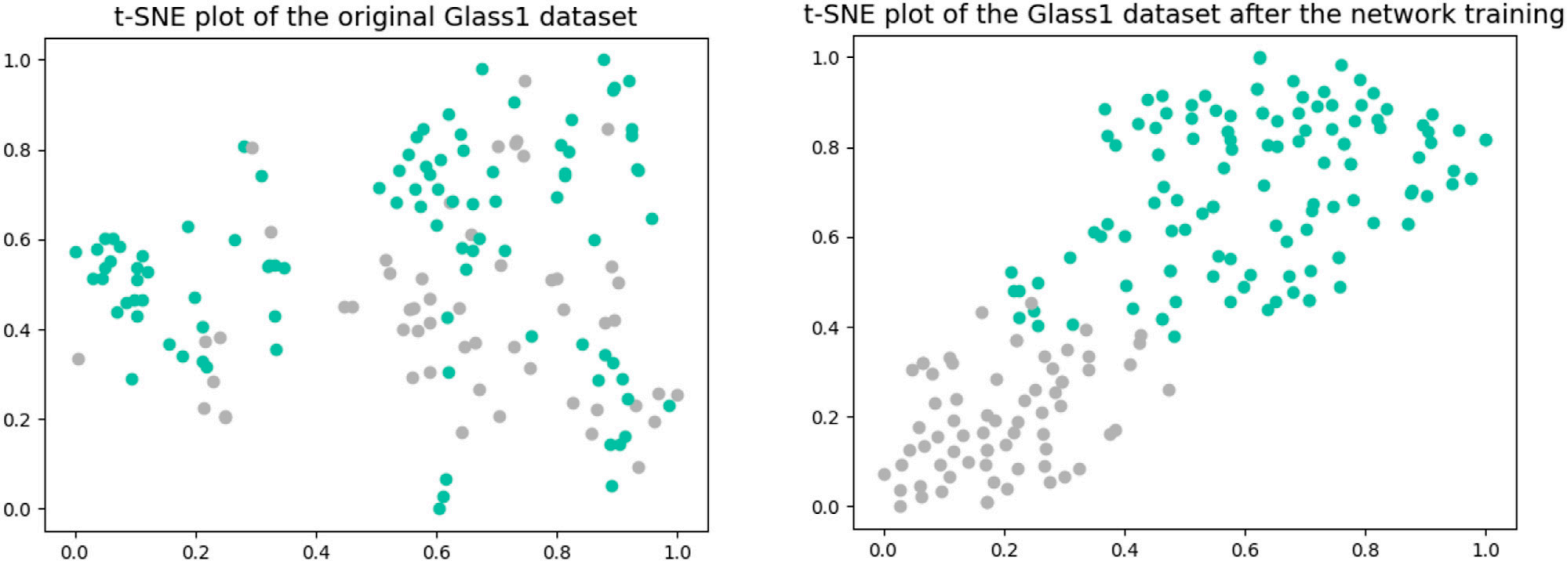
t-SNE plots of the original Glass1 dataset and after the network training.

When compared to the Gumbel distribution, the ReLU activation function shows some drawbacks for the class imbalance problem: it tends to underestimate the probability of minority nodes when dealing with the issue of class imbalance. Relative to the ReLU activation function, the Gumbel distribution function is not affected by the dying ReLU problem. Moreover, the Gumbel distribution is asymmetric, so that different penalties can be applied to the misclassification of both classes. In addition, the Gumbel distribution function is continuously differentiable, so it can be easily used as an activation function with optimization in a neural network. Finally, the whole DNN framework used for feature extraction is shown in Figure 2. The network used for feature extraction consists of three hidden layers, and we set the number of neurons in each layer to have the following relationship: the number of neurons in the next layer is half of the number of neurons in the previous layer. In the later experiments, we only set the number of neurons in the third layer, i.e., the dimension of the final embedding space, and the first two layers will make the corresponding changes according to the above rules. Figure 3 shows two t-SNE plots of the original Glass1 dataset and the dataset after the network training. It can be seen that after training, the different classes become easier to distinguish.

### Random Feature Oversampling Based on Center Distance

After obtaining the embedding space representation of samples, the data distribution is still imbalanced. The dataset in the embedding space is $X=\left\{x_{1},x_{2},\cdots ,x_{n}\right\}$ , n is the total number of samples, $x_{i}=\left[f_{i}^{1},f_{i}^{2},\cdots ,f_{i}^{p}\right]\in {\mathbb{R}}^{p}$ , i∈1,2,…,n . $f_{i}^{j}$ is the value of the sample $x_{i}$ on the j -th dimension feature, j∈1,2,…,p . For the minority class samples, the set of features in each dimension is denoted as $F=\left\{F^{1},F^{2},\cdots ,F^{p}\right\}$ , where $F^{j}=\left\{f_{1}^{j},f_{2}^{j},\cdots ,f_{n\_min}^{j}\right\}$ , j∈1,2,…,p . n_min is the number of the minority class samples. $F^{j}$ is the set of values of all minority class samples on the j -th dimension feature. The feature of each dimension of the new synthetic sample is randomly selected from the corresponding feature set, $x_{syn}=\left[f_{syn}^{1}\in F^{1},f_{syn}^{2}\in F^{2},\cdots ,f_{syn}^{p}\in F^{p}\right]$ .

**FIGURE 4:**
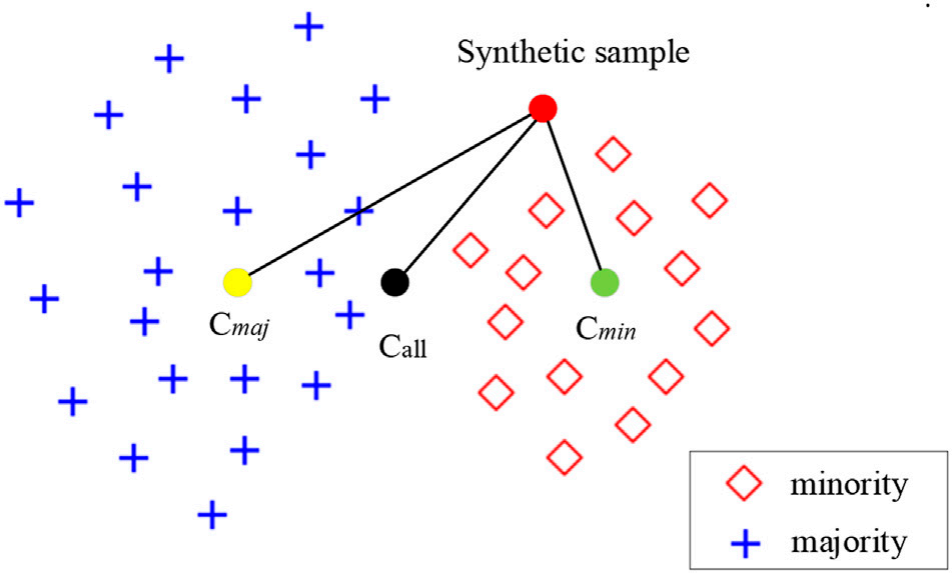
Validation of the new synthetic feature vector.

This method of randomly generated features can increase the diversity of the minority class samples and avoid overfitting. However, the method generates some outliers and noise, so a constraint based on class center distance is used to filter the synthetic samples. As shown in Figure 4, in the embedding space, the center of the majority class is $C_{maj}$ , the center of the minority class is $C_{min}$ , and the center of the whole data is $C_{all}$ . By calculating the distance between each center and the synthetic sample to determine whether the following equation is satisfied:

$$\begin{array}{c} d\left(x_{\mathrm{syn}},C_{\mathrm{maj}}\right)>d\left(x_{\mathrm{syn}},C_{\mathrm{all}}\right)>d\left(x_{\mathrm{syn}},C_{\min}\right) \end{array}$$ (3)

where d(⋅) is the distance function. If the synthesized sample fits this condition, it will be kept, otherwise, it will be deleted. In this paper, the influence of irregular data distribution is avoided by calculating the class centers in the embedding space. The number of synthesized samples is set to achieve balanced data distribution.

### Fuzzy Support Vector Machine

In many real-life applications, each sample has a different level of importance. For imbalanced data problems, the minority class samples are often more important than the majority class samples. In order to improve the classification performance, each sample needs to be assigned to a corresponding weight according to its importance. In this paper, a fuzzy support vector machine (FSVM) (Lin and Wang, 2002) is used as the classifier to achieve the assignment of different weights.

The data after sampling as $X=\left\{x_{1},x_{2},\cdots ,x_{n}\right\}$ , n is the total number of samples including all synthetic samples, $x_{i}\in {\mathbb{R}}^{p}$ , i∈1,2,…,n . p is the feature dimension. Assuming that the dataset is $D=\left\{\left(x_{1},y_{1}\right),\left(x_{2},y_{2}\right),\cdots ,\left(x_{n},y_{n}\right)\right\}$ . $y_{i}\in \left[1,-1\right]$ is the label of the corresponding sample. FSVM adds an attribute to each sample to expand the original data set to $D=\left\{\left(x_{1},y_{1},s_{1}\right),\left(x_{2},y_{2},s_{2}\right),\cdots ,\left(x_{n},y_{n},s_{n}\right)\right\}$ , $s_{i}\;$ represents the fuzzy membership value (MV) corresponding to different samples. The greater the value of s , the greater the importance of the sample. In this way, the optimization function of FSVM can be written as:

$$\begin{array}{c} \begin{array}{c} \min:\frac{1}{2}{∥w∥}^{2}+C\sum_{i=1}^{n}s_{i}\varepsilon_{i}\\ s.t.y_{i}\left(w*\phi \left(x_{i}\right)+b\right)\geq 1-\varepsilon_{i}\;\\ \varepsilon_{i}\geq 0 \end{array} \end{array}$$ (4)

where $w^{2}$ represents the margin ratio of the generalization ability of the learning model. The slack variable $\varepsilon_{i}$ represents the acceptable training error degree of the corresponding instance $x_{i}$ . C>0 is called the penalty parameter, it is a parameter that weighs the size of the separation interval and the number of misclassified points, as well as a trade-off between learning model accuracy and generalization ability. ϕ(⋅) is the mapping of high-dimensional feature space. The fuzzy membership value $s_{i}$ can adjust the punishment degree of the corresponding sample. In order to solve this optimization problem, firstly, Equation 4 is transformed into an unconstrained problem using the Lagrangian function:

$$\begin{array}{c} L\left(w,b,\alpha ,\beta \right)=\frac{1}{2}w^{2}+C\overset{n}{\underset{i=1}{\sum }}s_{i}\varepsilon_{i}-\overset{n}{\underset{i=1}{\sum }}\alpha_{i}\left(y_{i}\left(w*x_{i}+b\right)-1+\varepsilon_{i}\right)-\overset{n}{\underset{i=1}{\sum }}\beta_{i}\varepsilon_{i} \end{array}$$ (5)

The above formula satisfies the following conditions:

$$\begin{array}{c} \begin{array}{c} \frac{\partial L\left(w,b,\alpha ,\beta \right)}{\partial w}=w-\overset{n}{\underset{i=1}{\sum }}\alpha_{i}y_{i}x_{i}=0\\ \frac{\partial L\left(w,b,\alpha ,\beta \right)}{\partial b}=-\overset{n}{\underset{i=1}{\sum }}\alpha_{i}y_{i}=0\\ \frac{\partial L\left(w,b,\alpha ,\beta \right)}{\partial \varepsilon_{i}}=\varepsilon_{i}C-\alpha_{i}-\beta_{i}=0 \end{array} \end{array}$$ (6)

Introduce Equation 6 into Equation 5. The optimization problem is transformed into the following formula:

$$\begin{array}{c} \begin{array}{c} \min:-\overset{n}{\underset{i=1}{\sum }}\alpha_{i}+\frac{1}{2}\overset{n}{\underset{i=1}{\sum }}\overset{n}{\underset{j=1}{\sum }}y_{i}y_{j}\alpha_{i}\alpha_{j}\phi \left(x_{i}\right)\phi \left(x_{j}\right)\\ s.t.\overset{n}{\underset{i=1}{\sum }}y_{i}\alpha_{i}=0,\forall i:0\leq \alpha_{i}\leq s_{i}C \end{array} \end{array}$$ (7)

where $\alpha_{i}$ is the Lagrangian multiplier corresponding to $x_{i}$ , and it must also meet the KKT condition:

$$\begin{array}{c} \begin{array}{c} \forall i:\alpha_{i}\left(y_{i}\left(w*\phi \left(x_{i}\right)+b\right)-1+\varepsilon_{i}\right)=0\\ \forall i:\left(s_{i}C-\alpha_{i}\right)\varepsilon_{i}=0 \end{array} \end{array}$$ (8)

In this way, the value of $\alpha_{i}$ can be calculated, and w can be calculated according to the following formula:

$$\begin{array}{c} w=\overset{n}{\underset{j=1}{\sum }}\alpha_{i}\beta_{j}\phi \left(x_{i}\right) \end{array}$$ (9)

After that, the value of b can be calculated by Equation 8. The sample of $\alpha_{i}>0$ is called a support vector. When $0<\alpha_{i}<s_{i}C$ , the support vector is located on the boundary of the interval. When $\alpha_{i}=s_{i}C$ , the sample is located between the boundary of the interval and the separation hyperplane or on the side of the separation hyperplane that is misclassified. The biggest difference between traditional SVM and FSVM is that even though two samples have the same value of $\alpha_{i}$ , the two samples belong to different types of support vectors due to their different fuzzy membership values $s_{i}$ . Under normal circumstances, a smaller $s_{i}$ is assigned to the majority class to make the decision boundary more reasonable. Finally, the decision function of the optimal separating hyperplane can be expressed as:

$$\begin{array}{c} f\left(x\right)=sign\left(w*\phi \left(x_{i}\right)+b\right)=sign\left(\overset{n}{\underset{j=1}{\sum }}\alpha_{i}y_{i}\phi \left(x_{i}\right)\phi \left(x\right)+b\right) \end{array}$$ (10)

## Experiments and Results

### Evaluation Metrics and Datasets

In this paper, G-mean, F-measure and AUC values are used to comprehensively evaluate the classification quality of the model. In imbalanced classification, the overall accuracy is not effective in evaluating the classification results. To evaluate the imbalanced classification effect by accuracy may cause the model to be biased towards the majority class, because a high overall accuracy can be obtained by ensuring only the correct classification of the majority class. The overall accuracy ignores the important influence of the minority class.

F-measure is defined based on the metrics of Precision (Pre) and Sensitivity (Sen), which are defined as:

$$\begin{array}{c} Pre=\frac{TP}{TP+FP} \end{array}$$ (11)

$$\begin{array}{c} Sen=\frac{TP}{TP+FN} \end{array}$$ (12)

where TP (True Positives) denotes the number of positive observations (minority class) correctly classified as positive, FP (False Positives) denotes the number of negative observations (majority class) incorrectly classified as positive, FN (False Negatives) denotes the number of positive observations incorrectly classified as negative, and TN denotes the number of negative observations correctly classified as negative (Ye et al., 2020). The definition of F-measure is as

$$\begin{array}{c} F-\mathrm{measure}=2*Sen*Pre/\left(Sen+Pre\right) \end{array}$$ (13)

G-mean is defined based on the metrics of Sensitivity (Sen) and Specificity (Spe), which are defined as:

$$\begin{array}{c} Spe=\frac{TN}{TN+FP} \end{array}$$ (14)

$$\begin{array}{c} G-\mathrm{mean}=\sqrt{Sen*Spe} \end{array}$$ (15)

AUC (Area Under Curve) is defined as the area under the ROC curve and the coordinate axis. The value of this area will not be greater than 1. Among them, the ROC curve is called the receiver operating characteristic curve. It is based on a series of different binary classification methods (cutoff value or decision threshold), with the true positive rate (Sen) as the ordinate, and the false positive rate (1-Spe) is the curve drawn on the abscissa. The closer the AUC is to 1.0, the higher the authenticity of the detection method; when it is equal to 0.5, the authenticity is the lowest and it has no application value. The algorithm was tested on twelve binary classification datasets from the Keel database, as shown in Table 1.

**TABLE 1:** Description of the datasets.

Name	Attributes	Data size	Imbalance ratio
Cargood	6	1,728	24.04
Cleveland0vs4	13	177	12.62
Ecoli0147vs2356	7	336	10.59
Ecoli01vs235	7	244	9.17
Ecoli0267vs35	7	224	9.18
Glass1	9	214	1.82
Glass6	9	214	6.38
Pageblocks0	10	5,472	8.79
Vehicle3	18	846	2.99
Yeast1vs7	8	459	14.3
Yeast3	8	1,484	8.1
Yeast6	8	1,484	41.4

### Experiment Settings

In data feature processing, a deep neural network with four fully connected layers is used. When using fuzzy support vector machine for classification operation, the kernel function is Gaussian kernel function. For FSVM classifier, penalty constant C and the width of Gaussian kernel σ are selected by gird search method from the set $\left\{10^{-3},10^{-2},10^{-1},1,10^{1},10^{2},10^{3},10^{4}\right\}$ and $\left\{2^{-5},2^{-4},2^{-3},2^{-2},2^{-1},1,2^{1},2^{2},2^{3},2^{4}\right\}$ . The fuzzy membership value of the minority samples is set to the imbalanced ratio (IR), which is the ratio of the number of samples of the majority class to the number of the minority class in the data.

$$\begin{array}{c} IR=\frac{num_{maj}}{num_{min}} \end{array}$$ (16)

where $num_{min}$ is the number of the minority class samples, and $num_{maj}$ is the number of data of the majority class samples. The fuzzy membership value of the majority class is set to 1. In order to eliminate the randomness, five cross validation is applied, and the algorithms are executed for 5 independent runs.

In order to compare the classification performance of the proposed model, nine methods are used. *B-SMOTE* (Han et al., 2005) uses *SMOTE* (Chawla et al., 2002) to synthesize new samples for the minority-class samples lying around the boundary line. *SOMO* (Douzas and Bacao, 2017) produces a two-dimensional representation. Then it generates within-cluster and between-cluster synthetic samples. *KmeansSMOTE* (Douzas et al., 2018) uses k-means clustering algorithm and SMOTE to balance datasets, and only oversampling in safe areas to avoid noise. *FSVM-CEN* and *FSVM-HYP* (Batuwita and Palade, 2010) use a linear decay function to calculate the MVs based on the distance from the own class center or from the actual hyperplane, and finally use FSVM for classification. *FSVM-WD* and *FSVM-BD* (Yu et al., 2019) adopt a k-nearest neighbors-based probability density estimation to design a membership function based on the within-class and between-class relative density, and then assign weights to different samples. *ACFSVM* (Tao et al., 2020) first gives the corresponding formulation of affinity to calculate different affinities for each sample in the majority class. Then the class probability of each majority-class sample is determined using the kernel KNN technique and combined with its corresponding affinity as MVs. In addition, note that the parameters that existed in each algorithm adopt the default ones in each corresponding reference. For DFSVM, the margin *m* and the number of neurons in the third hidden layer of the deep neural network are selected by gird search method from the set {0.1, 0.2, 0.3, 0.4, 0.5, 0.6, 0.7, 0.8, 0.9} and {2,4,6,8,10,12}.

### Comparison of Imbalanced Classification Performance

To verify the effectiveness of the model on imbalance classification tasks, DFSVM is compared with nine representative and state-of-the-art class imbalance algorithms. Table 2 summarizes the experimental results on part of the datasets in terms of G-mean, F-measure and AUC, and highlights the best performing models in boldface. It can be observed that DFSVM achieves better performance in most cases. For the Cleveland0vs4 dataset, DFSVM improves 0.123 relative to ACFSVM under the G-mean metric. Although DFSVM does not achieve the best results on the AUC metric on the Cargood dataset, it achieves the best classification results on the G-mean and F-measure, which indicates that DFSVM has good recognition for the minority class. On the Pageblocks0 dataset, the classification performance of DFSVM did not achieve the best results on all three different evaluation metrics. However, its classification performance does not differ much from the best result. For example, for G-mean, it differs from the best by only 0.2%, and F-measure differs by 1%, which indicates that DFSVM still has a better classification effect. Yeast6 dataset has the highest imbalance ratio with IR = 41.4 and Glass1 dataset has the lowest imbalance ratio with IR = 1.82. The proposed method in this paper achieves better classification results on both Yeast6 and Glass1datasets. The best results were obtained for all evaluation metrics on the Yeast6 dataset, and two evaluation metrics for Glass1 dataset. This shows that DFSVM has good classification results for datasets with different imbalance ratios and it is robust.

**TABLE 2:** Results of different imbalanced classification methods on datasets.

Dataset	Cargood			Cleveland0vs4		
Measure	G-mean	F-measure	AUC	G-mean	F-measure	AUC
SMOTE	0.851 ± 0.027	0.716 ± 0.021	0.995 ± 0.001	0.373 ± 0.066	0.349 ± 0.063	0.962 ± 0.003
B-SMOTE	0.839 ± 0.007	0.693 ± 0.010	0.994 ± 0.000	0.420 ± 0.001	0.393 ± 0.000	0.963 ± 0.004
SOMO	0.864 ± 0.010	0.649 ± 0.035	0.988 ± 0.002	0.640 ± 0.087	0.553 ± 0.084	0.972 ± 0.012
KmeansSMOTE	0.881 ± 0.015	0.758 ± 0.033	0.993 ± 0.003	0.665 ± 0.169	0.549 ± 0.155	0.967 ± 0.007
FSVM-CEN	0.875 ± 0.023	0.616 ± 0.009	0.991 ± 0.003	0.778 ± 0.087	0.574 ± 0.051	0.982 ± 0.002
FSVM-HYP	0.886 ± 0.033	0.662 ± 0.011	0.991 ± 0.003	0.677 ± 0.058	0.541 ± 0.034	0.973 ± 0.001
FSVM-WD	0.776 ± 0.012	0.586 ± 0.039	0.993 ± 0.000	0.728 ± 0.055	0.615 ± 0.027	0.976 ± 0.002
FSVM-BD	0.848 ± 0.000	0.687 ± 0.000	**0.997 ± 0.000**	0.743 ± 0.003	0.662 ± 0.015	0.933 ± 0.003
ACFSVM	0.919 ± 0.011	0.774 ± 0.015	0.996 ± 0.001	0.816 ± 0.048	0.819 ± 0.022	0.985 ± 0.003
DFSVM	**0.940 ± 0.041**	**0.834 ± 0.050**	0.985 ± 0.019	**0.939 ± 0.028**	**0.824 ± 0.210**	**0.987 ± 0.005**
**Dataset**	**Ecoli0147vs2356**			**Ecoli01vs235**		
**Measure**	**G-mean**	**F-measure**	**AUC**	**G-mean**	**F-measure**	**AUC**
SMOTE	0.607 ± 0.038	0.469 ± 0.062	0.792 ± 0.019	0.834 ± 0.006	0.674 ± 0.020	0.927 ± 0.005
B-SMOTE	0.710 ± 0.005	0.622 ± 0.006	0.856 ± 0.013	0.867 ± 0.011	0.739 ± 0.017	**0.963 ± 0.003**
SOMO	0.762 ± 0.035	0.676 ± 0.024	0.898 ± 0.032	0.781 ± 0.011	0.690 ± 0.026	0.955 ± 0.009
KmeansSMOTE	0.743 ± 0.043	0.647 ± 0.033	0.914 ± 0.018	0.873 ± 0.008	0.741 ± 0.013	**0.963 ± 0.013**
FSVM-CEN	0.747 ± 0.029	0.597 ± 0.011	0.845 ± 0.056	0.787 ± 0.028	0.601 ± 0.046	0.932 ± 0.018
FSVM-HYP	0.654 ± 0.055	0.591 ± 0.031	0.829 ± 0.018	0.820 ± 0.004	0.711 ± 0.009	0.952 ± 0.005
FSVM-WD	0.683 ± 0.000	0.463 ± 0.000	0.801 ± 0.005	0.801 ± 0.007	0.634 ± 0.014	0.910 ± 0.011
FSVM-BD	0.686 ± 0.008	0.531 ± 0.011	0.799 ± 0.003	0.825 ± 0.023	0.576 ± 0.049	0.925 ± 0.005
ACFSVM	0.782 ± 0.024	0.533 ± 0.010	0.839 ± 0.013	0.817 ± 0.028	0.654 ± 0.037	0.924 ± 0.008
DFSVM	**0.852 ± 0.062**	**0.716 ± 0.029**	**0.930 ± 0.052**	**0.874 ± 0.058**	**0.809 ± 0.060**	0.955 ± 0.044
**Dataset**	**Ecoli0267vs35**			**Glass1**		
**Measure**	**G-mean**	**F-measure**	**AUC**	**G-mean**	**F-measure**	**AUC**
SMOTE	0.807 ± 0.025	0.621 ± 0.024	0.937 ± 0.014	0.682 ± 0.008	0.634 ± 0.010	0.734 ± 0.020
B-SMOTE	0.771 ± 0.028	0.641 ± 0.027	0.915 ± 0.027	0.702 ± 0.008	0.606 ± 0.011	0.790 ± 0.016
SOMO	0.825 ± 0.012	0.735 ± 0.012	0.929 ± 0.021	0.678 ± 0.003	0.612 ± 0.002	0.713 ± 0.010
KmeansSMOTE	0.855 ± 0.021	0.702 ± 0.010	0.949 ± 0.005	0.755 ± 0.012	**0.689 ± 0.017**	0.759 ± 0.015
FSVM-CEN	0.765 ± 0.037	0.611 ± 0.007	0.906 ± 0.025	0.611 ± 0.011	0.583 ± 0.005	0.707 ± 0.033
FSVM-HYP	0.822 ± 0.004	0.676 ± 0.017	0.922 ± 0.032	0.643 ± 0.016	0.588 ± 0.018	0.708 ± 0.015
FSVM-WD	0.848 ± 0.018	0.615 ± 0.016	0.939 ± 0.005	0.598 ± 0.046	0.575 ± 0.020	0.732 ± 0.009
FSVM-BD	0.836 ± 0.010	0.647 ± 0.020	0.939 ± 0.012	0.638 ± 0.025	0.584 ± 0.030	0.706 ± 0.007
ACFSVM	0.869 ± 0.011	0.711 ± 0.005	0.923 ± 0.029	0.661 ± 0.005	0.636 ± 0.005	0.683 ± 0.015
DFSVM	**0.922 ± 0.012**	**0.764 ± 0.069**	**0.988 ± 0.004**	**0.692 ± 0.059**	0.629 ± 0.032	**0.792 ± 0.026**
**Dataset**	**Glass6**			**Pageblocks0**		
**Measure**	**G-mean**	**F-measure**	**AUC**	**G-mean**	**F-measure**	**AUC**
SMOTE	0.901 ± 0.014	0.808 ± 0.025	0.974 ± 0.002	0.890 ± 0.003	0.735 ± 0.011	0.960 ± 0.001
B-SMOTE	0.884 ± 0.033	0.818 ± 0.045	0.983 ± 0.006	0.903 ± 0.001	0.754 ± 0.002	0.963 ± 0.001
SOMO	0.861 ± 0.018	0.747 ± 0.019	0.969 ± 0.003	0.852 ± 0.003	0.746 ± 0.017	0.959 ± 0.005
KmeansSMOTE	0.878 ± 0.021	0.808 ± 0.022	0.967 ± 0.011	0.903 ± 0.009	**0.815 ± 0.014**	0.971 ± 0.001
FSVM-CEN	0.862 ± 0.017	0.760 ± 0.031	0.971 ± 0.005	0.908 ± 0.004	0.711 ± 0.001	0.960 ± 0.007
FSVM-HYP	0.910 ± 0.012	0.823 ± 0.009	0.982 ± 0.004	0.898 ± 0.001	0.746 ± 0.003	**0.975 ± 0.002**
FSVM-WD	0.892 ± 0.010	0.810 ± 0.018	0.978 ± 0.001	0.874 ± 0.001	0.485 ± 0.001	0.962 ± 0.002
FSVM-BD	0.891 ± 0.016	0.810 ± 0.006	0.983 ± 0.009	0.916 ± 0.003	0.587 ± 0.004	0.970 ± 0.010
ACFSVM	0.904 ± 0.011	0.777 ± 0.034	0.950 ± 0.007	**0.917 ± 0.001**	0.782 ± 0.001	0.961 ± 0.001
DFSVM	**0.939 ± 0.044**	**0.831 ± 0.044**	**0.989 ± 0.009**	0.915 ± 0.021	0.805 ± 0.023	0.960 ± 0.016
**Dataset**	**Vehicle3**			**Yeast1vs7**		
**Measure**	**G-mean**	**F-measure**	**AUC**	**G-mean**	**F-measure**	**AUC**
SMOTE	0.741 ± 0.001	0.631 ± 0.001	0.863 ± 0.001	0.418 ± 0.024	0.223 ± 0.013	0.697 ± 0.010
B-SMOTE	0.746 ± 0.016	0.634 ± 0.024	0.865 ± 0.017	0.435 ± 0.005	0.244 ± 0.012	0.717 ± 0.006
SOMO	0.766 ± 0.010	0.676 ± 0.013	0.899 ± 0.008	0.509 ± 0.120	0.433 ± 0.101	0.745 ± 0.010
KmeansSMOTE	0.787 ± 0.005	0.694 ± 0.010	0.903 ± 0.007	0.555 ± 0.054	0.466 ± 0.045	0.748 ± 0.003
FSVM-CEN	0.831 ± 0.007	0.712 ± 0.009	0.906 ± 0.003	0.611 ± 0.042	0.359 ± 0.028	0.817 ± 0.022
FSVM-HYP	0.804 ± 0.004	0.699 ± 0.004	0.904 ± 0.003	0.668 ± 0.041	0.449 ± 0.017	0.816 ± 0.004
FSVM-WD	0.761 ± 0.002	0.622 ± 0.003	0.852 ± 0.003	0.627 ± 0.000	0.258 ± 0.000	0.740 ± 0.000
FSVM-BD	0.774 ± 0.020	0.638 ± 0.018	0.862 ± 0.014	0.662 ± 0.011	0.286 ± 0.007	0.776 ± 0.006
ACFSVM	0.821 ± 0.008	0.725 ± 0.012	**0.934 ± 0.004**	0.659 ± 0.009	0.333 ± 0.005	0.763 ± 0.012
DFSVM	**0.846 ± 0.026**	**0.737 ± 0.034**	0.884 ± 0.026	**0.697 ± 0.077**	**0.476 ± 0.028**	**0.819 ± 0.074**
**Dataset**	**Yeast3**			**Yeast6**		
**Measure**	**G-mean**	**F-measure**	**AUC**	**G-mean**	**F-measure**	**AUC**
SMOTE	0.837 ± 0.004	0.683 ± 0.001	0.933 ± 0.006	0.625 ± 0.001	0.342 ± 0.011	0.880 ± 0.003
B-SMOTE	0.831 ± 0.002	0.672 ± 0.001	0.932 ± 0.002	0.703 ± 0.056	0.529 ± 0.051	0.911 ± 0.001
SOMO	0.851 ± 0.000	0.775 ± 0.001	**0.970 ± 0.002**	0.593 ± 0.053	0.451 ± 0.047	0.857 ± 0.012
KmeansSMOTE	0.863 ± 0.015	0.749 ± 0.027	0.964 ± 0.004	0.655 ± 0.014	0.443 ± 0.021	0.726 ± 0.015
FSVM-CEN	0.905 ± 0.005	0.720 ± 0.003	0.964 ± 0.003	0.849 ± 0.027	0.374 ± 0.025	0.921 ± 0.002
FSVM-HYP	0.896 ± 0.006	0.720 ± 0.002	0.959 ± 0.001	0.593 ± 0.016	0.365 ± 0.021	0.905 ± 0.004
FSVM-WD	0.891 ± 0.000	0.622 ± 0.000	0.957 ± 0.000	0.813 ± 0.001	0.287 ± 0.000	0.915 ± 0.001
FSVM-BD	0.910 ± 0.001	0.762 ± 0.002	0.974 ± 0.001	0.801 ± 0.072	0.339 ± 0.005	0.922 ± 0.005
ACFSVM	0.901 ± 0.003	0.778 ± 0.005	0.945 ± 0.003	0.805 ± 0.001	0.412 ± 0.007	0.917 ± 0.001
DFSVM	**0.917 ± 0.030**	**0.781 ± 0.040**	0.968 ± 0.033	**0.856 ± 0.058**	**0.501 ± 0.059**	**0.935 ± 0.064**

The bold values mean the best results.

In addition, statistical tests were performed in this paper to verify the validity and significance of the proposed method. Under the null hypothesis, all algorithms are equivalent (i.e., any difference between their mean ranks is only random). The Friedman statistic

$$\begin{array}{c} \chi_{F}^{2}=\frac{12N}{k\left(k+1\right)}\left[\overset{k}{\underset{j=1}{\sum }}R_{j}^{2}-\frac{k{\left(k+1\right)}^{2}}{4}\right] \end{array}$$ (17)

is distributed according to the $\chi_{F}^{2}$ distribution with k−1 degrees of freedom when *N* and *k* are large enough. *N* is the number of datasets, *k* is the number of algorithms performed for comparison, and *R* is the average ranking of the algorithms under different datasets. Friedman’s $\chi_{F}^{2}$ statistic is undesirably conservative, so we use a better statistic

$$\begin{array}{c} F_{F}=\frac{\left(N-1\right)\chi_{F}^{2}}{N\left(k-1\right)-\chi_{F}^{2}} \end{array}$$ (18)

which is distributed according to the *F*-distribution with k−1 and  (k−1)(N−1) degrees of freedom. We calculated the average ranking of different algorithms under the G-mean metric based on the experimental results in Table 2, and calculated the $F_{F}=8.055$ . At significance level α=0.05 , the critical value is 1.976. Since $F_{F}$ under G-mean is greater than 1.976, the null hypothesis is rejected, i.e., the algorithms compared are not equivalent at α=0.05 . Since the null hypothesis has been rejected, the Nemenyi post-hoc test is utilized to complete the performance analysis, and DFSVM is regarded as the control method. When the difference between the mean rankings is greater than the critical difference ( $CD=q_{\alpha }\sqrt{k\left(k+1\right)/6N}=3.91$ , where $q_{\alpha }=3.163$ in this paper), they are considered significantly different. The average ranking of the comparison method and DFSVM and the differences between them are given in Table 3. As can be seen from Table 3, DFSVM outperforms the other algorithms. Although it is not significantly different from KmeansSMOTE, FSVM-BD and ACFSVM, DFSVM is better than them in terms of classification effect on different data sets.

**TABLE 3:** Average ranking of different methods on G-mean and its ranking difference with DFSVM.

Method	Average rank	Difference
SMOTE	7.67	6.34
B-SMOTE	7.00	5.67
SOMO	7.50	6.17
KmeansSMOTE	5.17	3.84
FSVM-CEN	5.50	4.17
FSVM-HYP	5.67	4.34
FSVM-WD	6.75	5.42
FSVM-BD	5.08	3.75
ACFSVM	3.33	2.00
DFSVM	1.33	N/A

### Influence of Activation Function on the Performance of the Proposed Method

**FIGURE 5:**
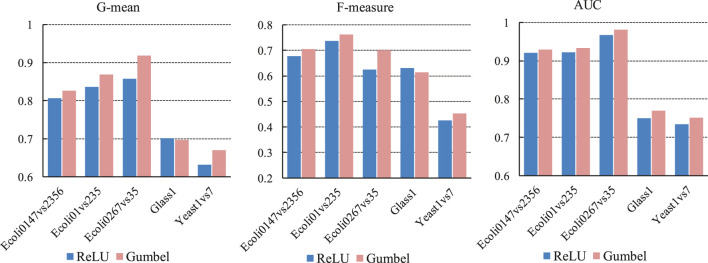
Classification effect of DFSVM under two different activation functions.

In this subsection, we compare the effect of the Gumbel activation function and the ReLU activation function on the experimental results. We selected five datasets, three of them are Ecoli datasets. They are different from each other, for example, in the Ecoli0147vs2356 dataset, positive samples belong to classes 0, 1, 4, 7 and negative samples belong to classes 2, 3, 5, 6; in the Ecoli01vs235 dataset, positive class samples belong to classes 0, 1 and negative samples belong to classes 2, 3, 5. In the experiments, the structure of the deep neural network is fixed, the number of neurons in the third hidden layer is set to 8, and the margin of triplet loss is set to 0.2. The experimental results are shown by Figure 5. It can be seen that the classification effect of Gumbel function is better than ReLU function. In the Ecoli0267vs35 dataset, the Gumbel function showed the most significant improvement. With the G-mean and F-measure metrics, the classification effect of the Gumbel function is 6.19 and 7.71% higher than the ReLU function. On the Glass1 dataset, although the classification quality of the Gumbel function is not as good as that of the ReLU function in the G-mean and F-measure metrics, the Gumbel function achieves better results in the AUC metric.

### Influence of Classifier and Sampling Algorithms on Classification Performance

This subsection compares the classification quality of two different classifiers, SVM and FSVM. We also selected 5 datasets. In the experiments, the structure of the deep neural network is fixed, the number of neurons in the third hidden layer is also set to 8, and the margin of triplet loss is set to 0.2. The experimental results are shown in Table 4, and highlights the best performing models in boldface. It can be seen that the classification quality of FSVM is better than that of SVM. On the Ecoli01vs235 dataset, the AUC values of FSVM are slightly lower than those of SVM, but FSVM is 0.0524 and 0.2428 higher than SVM in G-mean and F-measure metrics. FSVM assigns a higher misclassification cost to the minority class in the objective function and therefore has a better imbalance classification effect.

**TABLE 4:** Classification effect of DFSVM under two different base classifiers.

Evaluation metrics	G-mean		F-measure		AUC	
Dataset	FSVM	SVM	FSVM	SVM	FSVM	SVM
Ecoli0147vs2356	**0.8260 ± 0.0490**	0.8082 ± 0.0748	**0.7060 ± 0.0836**	0.6868 ± 0.1072	**0.9284 ± 0.0502**	0.9178 ± 0.0351
Ecoli01vs235	**0.8687 ± 0.0950**	0.8163 ± 0.1152	**0.7628 ± 0.1594**	0.5200 ± 0.1618	0.9334 ± 0.0353	**0.9545 ± 0.0215**
Ecoli0267vs35	**0.9187 ± 0.0647**	0.7986 ± 0.0883	**0.7412 ± 0.1162**	0.7270 ± 0.1109	**0.9814 ± 0.0191**	0.9698 ± 0.0305
Glass1	**0.6971 ± 0.0463**	0.6667 ± 0.0622	**0.6134 ± 0.0591**	0.5818 ± 0.0787	**0.7699 ± 0.0421**	0.7446 ± 0.0544
Yeast1vs7	**0.6705 ± 0.0862**	0.5029 ± 0.1372	**0.4539 ± 0.1453**	0.2630 ± 0.1013	**0.7508 ± 0.0538**	0.6655 ± 0.0921

The bold values mean the best results.

In addition, we compare the center distance based random feature oversampling method in DFSVM with different sampling methods, such as SMOTE, B-SMOTE, SOMO and KmeansSMOTE. In the experiments, we only replace the different sampling methods and keep the remaining settings the same. The experiments were conducted on four different datasets, and the results are shown in Table 5, which shows that the random feature oversampling method based on the center distance is better than the other sampling methods. The best results under each dataset are bolded. Although DFSVM does not have the best classification results on the G-mean and AUC for the Ecoli01vs235 dataset, it achieves the best classification results on the F-measure.

**TABLE 5:** The experimental results of different sampling methods.

Dataset	Ecoli0147vs2356			Ecoli01vs235		
Measure	G-mean	F-measure	AUC	G-mean	F-measure	AUC
SMOTE	0.7273 ± 0.0456	0.6363 ± 0.1203	0.8354 ± 0.0379	0.8384 ± 0.0461	0.7368 ± 0.0972	0.9321 ± 0.0691
B-SMOTE	0.7323 ± 0.0771	0.5517 ± 0.1605	0.9017 ± 0.0278	0.8752 ± 0.0895	0.6956 ± 0.1396	**0.9627 ± 0.0133**
SOMO	0.7911 ± 0.1964	0.6823 ± 0.1817	0.9167 ± 0.0336	0.8719 ± 0.0502	0.6778 ± 0.0896	0.9616 ± 0.0216
KmeansSMOTE	0.8129 ± 0.1817	0.6933 ± 0.2175	0.9203 ± 0.0435	**0.8913 ± 0.0548**	0.7320 ± 0.0861	0.9271 ± 0.0498
DFSVM	**0.8260 ± 0.0490**	**0.7060 ± 0.0836**	**0.9284 ± 0.0502**	0.8687 ± 0.0950	**0.7628 ± 0.1594**	0.9334 ± 0.0353
**Dataset**	**Ecoli0267vs35**			**Yeast1vs7**		
**Measure**	**G-mean**	**F-measure**	**AUC**	**G-mean**	**F-measure**	**AUC**
SMOTE	0.8665 ± 0.0964	0.7125 ± 0.1219	0.9528 ± 0.0366	0.4941 ± 0.1579	0.3363 ± 0.0838	0.6857 ± 0.1466
B-SMOTE	0.8244 ± 0.0994	0.4516 ± 0.1251	0.8029 ± 0.0352	0.5135 ± 0.1213	0.3533 ± 0.1079	0.7308 ± 0.0669
SOMO	0.8620 ± 0.0142	0.6897 ± 0.1330	0.9729 ± 0.0074	0.5811 ± 0.1708	0.4242 ± 0.1174	0.6723 ± 0.0681
KmeansSMOTE	0.8967 ± 0.1108	0.7181 ± 0.1567	0.9611 ± 0.0350	0.5261 ± 0.1961	0.2285 ± 0.0734	0.6652 ± 0.0693
DFSVM	**0.9187 ± 0.0647**	**0.7412 ± 0.1162**	**0.9814 ± 0.0191**	**0.6705 ± 0.0862**	**0.4539 ± 0.1453**	**0.7508 ± 0.0538**

The bold values mean the best results.

### Influence of Network Structure and Margin on the Performance of the Proposed Method

**FIGURE 6:**
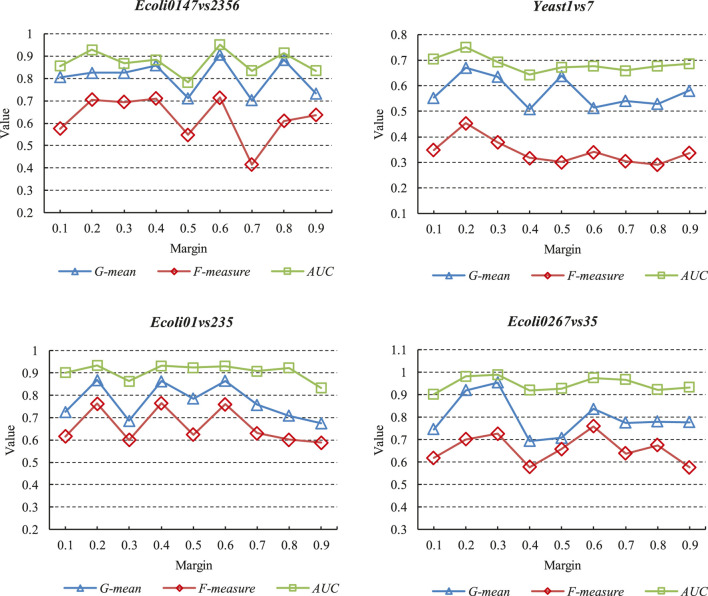
Classification results under different margins.

This subsection conducts comparative experiments on different network structures of DFSVM. First, we selected three Ecoli datasets and one Yeast dataset for experiments on different margins. In the experiments, the margin of triplet loss is taken from {0.1, 0.2, 0.3, 0.4, 0.5, 0.6, 0.7, 0.8, 0.9} and the number of neurons of the third hidden layer of the deep neural network is set to 8. The experimental results on the evaluation metrics are shown in Figure 6. The x-axis represents the values taken for different margins, and the y-axis represents the experimental result values for different metrics, such as G-mean, F-measure and AUC. It can be seen that in the same dataset, different metrics have the same trend with margin. However, the tendency of variation is not the same among different datasets. On the Ecoli0267vs35 dataset, the best classification results are obtained when the margin is 0.3. However, on the Ecoli01vs235 dataset, the classification is worse when the margin is 0.3. For all datasets, a smaller margin gives a better imbalanced classification result. Smaller margins make it easier for the triplet loss to converge to zero, but too small margins can make it difficult for the model to distinguish similar features. We also conducted comparison experiments on DFSVM with different hidden layer sizes. The fixed margin size in the experiments is 0.2, and the number of neurons in the third hidden layer of the neural network is taken from {2,4,6,8,10,12}. The experimental results under different evaluation metrics are shown in Tables 6–8. We selected three Ecoli datasets and three Yeast datasets, and the best results are bolded. It can be found that for the Ecoli dataset, the better results are achieved when the number of nodes is 6 and 8. For the Yeast dataset, better classification quality is achieved at node numbers of 8 and 10. In addition, it can be seen from Table 2 that the features extracted by the deep neural network are more beneficial for classification in comparison with other FSVM-based methods.

**TABLE 6:** Classification results of DFSVM with different number of neurons for G-mean.

*Dataset*	12	10	8	6	4	2
*Ecoli0147vs2356*	0.8339 ± 0.0561	0.7289 ± 0.2079	0.8434 ± 0.0691	**0.8490 ± 0.0623**	0.7968 ± 0.0697	0.7419 ± 0.0807
*Ecoli01vs235*	0.7964 ± 0.0763	0.8186 ± 0.0688	**0.8738 ± 0.0579**	0.7892 ± 0.0693	0.8233 ± 0.0610	0.8626 ± 0.0372
*Ecoli0267vs35*	0.7585 ± 0.0948	0.7370 ± 0.0810	**0.9150 ± 0.0119**	0.7902 ± 0.0622	0.8626 ± 0.0871	0.7655 ± 0.0882
*Yeast1vs7*	0.5690 ± 0.0669	0.6145 ± 0.0730	0.6106 ± 0.1270	**0.6969 ± 0.0776**	0.6026 ± 0.0648	0.6062 ± 0.2919
*Yeast3*	0.8058 ± 0.0417	0.8378 ± 0.0285	0.8700 ± 0.0291	0.8883 ± 0.0240	**0.8977 ± 0.0301**	0.8855 ± 0.0272
*Yeast6*	0.5893 ± 0.0505	**0.8022 ± 0.0589**	0.6220 ± 0.1238	0.5678 ± 0.0811	0.7785 ± 0.0924	0.6239 ± 0.0877

The bold values mean the best results.

**TABLE 7:** Classification results of DFSVM with different number of neurons for F-measure.

*Dataset*	12	10	8	6	4	2
*Ecoli0147vs2356*	0.7110 ± 0.1244	0.3965 ± 0.1425	**0.7116 ± 0.1055**	0.4622 ± 0.1293	0.3637 ± 0.1157	0.4010 ± 0.0858
*Ecoli01vs235*	0.7721 ± 0.0889	0.6922 ± 0.1053	**0.8068 ± 0.0603**	0.6703 ± 0.0877	0.7139 ± 0.0848	0.7903 ± 0.0886
*Ecoli0267vs35*	0.7196 ± 0.1114	0.6994 ± 0.1006	**0.7271 ± 0.1246**	0.6795 ± 0.0836	0.6521 ± 0.1422	0.6118 ± 0.1266
*Yeast1vs7*	0.2817 ± 0.0769	**0.3352 ± 0.0799**	0.3037 ± 0.1077	0.2417 ± 0.0775	0.2412 ± 0.0665	0.1067 ± 0.0675
*Yeast3*	0.6403 ± 0.0580	0.6837 ± 0.0440	0.7000 ± 0.0469	0.6832 ± 0.0394	**0.7516 ± 0.0402**	0.5284 ± 0.0727
*Yeast6*	0.3611 ± 0.0415	**0.4005 ± 0.0596**	0.2980 ± 0.0927	0.3535 ± 0.0668	0.3776 ± 0.0646	0.3295 ± 0.0635

The bold values mean the best results.

**TABLE 8:** Classification results of DFSVM with different number of neurons for AUC.

*Dataset*	12	10	8	6	4	2
*Ecoli0147vs2356*	0.8879 ± 0.0641	0.7467 ± 0.1766	0.9003 ± 0.0754	**0.9303 ± 0.0521**	0.8155 ± 0.0916	0.7442 ± 0.0918
*Ecoli01vs235*	0.9024 ± 0.0599	**0.9467 ± 0.0149**	0.9353 ± 0.0440	0.9394 ± 0.0413	0.9370 ± 0.0605	0.9151 ± 0.0446
*Ecoli0267vs35*	0.9451 ± 0.0290	0.9363 ± 0.0413	**0.9855 ± 0.0044**	0.9067 ± 0.0508	0.9533 ± 0.0421	0.8874 ± 0.0976
*Yeast1vs7*	0.6386 ± 0.0884	**0.7562 ± 0.0663**	0.7298 ± 0.1079	0.7232 ± 0.0987	0.6409 ± 0.1111	0.7362 ± 0.1286
*Yeast3*	0.8886 ± 0.0414	0.9364 ± 0.0228	0.9429 ± 0.0223	0.9373 ± 0.0220	0.8978 ± 0.0333	**0.9461 ± 0.0217**
*Yeast6*	0.7485 ± 0.0454	**0.8855 ± 0.0640**	0.7555 ± 0.0923	0.6590 ± 0.0688	0.8522 ± 0.0864	0.7045 ± 0.0848

The bold values mean the best results.

## Conclusion

This paper proposes an imbalanced classification method combined with deep neural networks, DFSVM. In order to obtain features with intra-class similarity and inter-class discrimination, a deep neural network is trained using triplet loss function and Gumbel activation function to obtain the deep feature representation. The results of the experiments show that the proposed feature extraction method has good information acquisition ability and can effectively distinguish different classes. To balance the data distribution, a random feature sampling algorithm based on the center of class is used in the minority samples to maintain the diversity of the minority class samples. Compared with other sampling algorithms, it can effectively avoid overfitting and improve the generalization performance of the model. Fuzzy support vector machine has provided a higher misclassification loss for the minority class, and it enhanced the classification performance of the algorithm for the minority class. According to the experimental results, it can be found that the proposed DFSVM has good classification results on evaluation metrics: G-means, F-measure, and AUC. In future work, more efficient network structures and more robust feature extractors can be used to provide valid measures for imbalanced classification.

## Data Availability

Publicly available datasets were analyzed in this study. This data can be found here: https://sci2s.ugr.es/keel/imbalanced.php.
